# NDUFC1 Is Upregulated in Gastric Cancer and Regulates Cell Proliferation, Apoptosis, Cycle and Migration

**DOI:** 10.3389/fonc.2021.709044

**Published:** 2021-12-13

**Authors:** Liang Xu, Xiuxiu Chen, Hongtao Jiang, Jian Xu, Lixia Wang, Yuemin Sun

**Affiliations:** ^1^ Department of Organ Transplantation, The Second Affiliated Hospital of Hainan Medical University, Hainan, China; ^2^ Surgery of Breast Nail, The Second Affiliated Hospital of Hainan Medical University, Hainan, China; ^3^ Department of Pancreatic & Gastric Surgery, National Cancer Center/National Clinical Research Center for Cancer/Cancer Hospital, Chinese Academy of Medical Sciences and Peking Union Medical College, Beijing, China

**Keywords:** gastric cancer, NDUFC1, cell proliferation, cell apoptosis, cell migration

## Abstract

Gastric cancer is one of the most common primary tumors of the digestive system. NADH: ubiquinone oxidoreductase subunit C1 (NDUFC1), which is an accessory subunit of the NADH dehydrogenase (complex I), is responsible for the transportation of electrons from NADH to the respiratory chain essential for the oxidative phosphorylation. However, little is known about the roles of NDUFC1 in carcinogenesis. In this study, NDUFC1 protein level in NSCLC tissues was tested by immunohistochemistry (IHC) staining. NDUFC1 mRNA level in gastric cancer cell lines was determined by qRT-PCR. MGC-803 and SGC-7901 cells were transfected with shNDUFC1 lentivirus designed to silence NDUFC1. MTT assay, CCK8 assay, wound healing assay and transwell migration assay were conducted. Cell cycle and apoptosis were detected by flow cytometry. *In vivo* experiments were performed using nude mice. The results indicated that overexpressed NDUFC1 in gastric cancer was related to more serious tumor infiltrates, a higher risk of lymphatic metastasis, a higher proportion of positive lymph nodes, and a more advanced tumor stage. Compared with shCtrl groups, MGC-803 and SGC-7901 of shNDUFC1 groups had lower abilities of proliferation and migration, higher levels of apoptosis. NDUFC1 knockdown also inhibited SGC-7901 cell growth *in vivo* and suppressed Ki67 expression in xenograft tumors. More importantly, we found that NDUFC1 downregulation made the levels of P-Akt, P-mTOR, CCND1, CDK6, PIK3CA, Bcl-2, Survivin, and XIAP decreased, and that PI3K/AKT signaling pathway agonist SC79 rescued the inhibitory effects on cell proliferation and migration, reversed the promoted effects on cell apoptosis caused by NDUFC1 knockdown. More importantly, compared with NDUFC1 knockdown group, the expression of P-Akt, Bcl-2, Survivin, and XIAP was raised in shNDUFC1 + SC79 group. Thus, our suspicion was that NDUFC1 exacerbates NSCLC progression *via* PI3K/Akt pathway. Taken together, our study indicated that targeting NDUFC1 could open innovative perspectives for new multi-targeting approaches in the treatment of gastric cancer.

## Introduction

Gastric cancer is one of the most commonly diagnosed malignant tumors originating from the digestive system (1, 2). Globally, gastric cancer is the third leading cause of cancer-related deaths, with a very low 5-year survival rate and poor prognosis (1). Although surgical treatment has a good therapeutic effect on patients with early gastric cancer, due to the low early diagnosis rate, most patients are diagnosed with advanced gastric cancer, against which pharmaceutical therapy is the major strategy of treatment (3, 4). Recently, molecular targeted therapy has been considered as one of the most promising treatments for gastric cancer, which is facilitated by the development of molecular mechanisms of malignant biological behaviors such as proliferation, growth, invasion, and metastasis of gastric cancer (5–8). Till now, several molecular targeted drugs such as cetuxiumab (targeting EGFR) (9), trastuzumab (targeting HER2), and bevacizumab (targeting VEGF) have been used in the treatment of gastric cancer (5). However, the prognosis of gastric cancer is still poor because of the limited understanding of gastric cancer occurrence and development, which is a complex procedure involving multi-gene and multi-factor (10, 11). Therefore, the in-depth study of the molecular mechanism of gastric cancer could contribute to develop novel therapeutic target and improve pharmaceutical efficiency of gastric cancer.

NADH dehydrogenase is one of the two key rate-limiting enzymes in the de-electronization of substrates, whose activity determines the oxidation efficiency of substrates (12). Physiological studies have shown that NADH dehydrogenase plays a key role in mitochondrial energy production and metabolism through regulation of electron transport chain of mitochondria (13, 14). NADH: ubiquinone oxidoreductase subunit C1 (NDUFC1), which is an accessory subunit of the NADH dehydrogenase (complex I), is responsible for the transportation of electrons from NADH to the respiratory chain essential for the oxidative phosphorylation (15). Adjaye reported that NDUFC1 may be involved in the OCT4-regulated activation of the poised OXPHOS machinery during his study of human embryonic stem cells and human embryonal carcinoma cells (16). However, the function of NDUFC1 and its relationship with human diseases, especially cancer, are rarely reported and still largely unknown.

In this study, we presented the first report of the relationship between NDUFC1 expression and development of gastric cancer. The expression levels of NDUFC1 in tumor tissues of 88 patients with gastric cancer were detected, compared with normal tissues, and statistically analyzed with tumor characteristics. Human gastric cancer cell lines with NDUFC1 knockdown were constructed to reveal the effects of NDUFC1 depletion on cell proliferation, cell apoptosis, cell cycle, and cell migration of gastric cancer cells. Mice xenograft model was further utilized to verify the inhibition of gastric cancer by NDUFC1 knockdown *in vivo*. Therefore, we identified NDUFC1 as a tumor promotor in gastric cancer, which may be a promising therapeutic target for treatment of gastric cancer.

## Material and Methods

### Cell Culture

Human gastric cancer cell lines AGS, MGC-803, and SGC-7901were purchased from BeNa Technology (Hangzhou, Zhejiang, China). AGS cells were cultured in 90% F-12K medium with 10% fetal bovine serum (FBS). MGC-803 cells were maintained in DMEM medium with 10% FBS. SGC-7901 cells were grown in RPMI-1640 medium with 10% FBS. All culture medium was changed every 2–3 days and cells were humid cultured in a 37°C 5% CO_2_ incubator.

### Immunohistochemistry

High density human gastric cancer and para-cancerous tissue microarray, and also 88 cancer tissues and 87 para-cancerous tissues, were prepared for immunohistochemistry. The tissues of patients with gastric cancer were collected from 2,012.12 to 2,015.8 during the operation and the tissues were embedded with paraffin. Each tissue specimen was cut into 5 μm section. Patients’ information and related data were collected as well. Written informed consent was provided by each patient before the operation.

Before immunohistochemistry assay, the microarray was baked at 60°C for 30 min. After dehydrated in xylene and rehydrated in graded alcohol, citric acid buffer was added for antigen retrieval (120°C for 20 min). The endogenous peroxidase was blocked with 3% H_2_O_2_ for 10 min. For immunohistochemical staining, a rabbit polyclonal antibody to NDUFC1 antibody at 1:50 on tissue sections at 4°C overnight. After washing, the secondary antibody was added and incubated for 2 h at room temperature. DAB color was developed with diaminobenzene for 10 min, then all slices were counterstained with hematoxylin. Slides were pictured with microscopic and viewed with ImageScope and CaseViewer. All slides were examined randomly by two independent pathologists. Staining percentage scores were classified as: 1 (1–24%), 2 (25–49%), 3 (50–74%), and 4 (75–100%). Staining intensity was scored as 0 (Signalless color), 1 (brown), 2 (light yellow), and 3 (dark brown). IHC outcomes were determined by percentage and intensity scores. Antibodies used here were shown in **Table S1**.

### Plasmid Construction, Lentivirus Infection and Cell Transfection

Short hairpin RNA sequences against human NDUFC1 gene were designed by Shanghai Bioscienceres (Shanghai, China) as follows: 5’-AAAGAAGAAATGGGCTGGAAT-3’, 5’-GTGGATCTATCTCATCAAACA-3’, and 5’-ATCAAAGTTCTACGTGCGAGA-3’. Preparation of double-stranded DNA oligo was accomplished by Jierui Co., Ltd (Shanghai, China) and ligated with linearized BR-V-108 Vector using Fermentas T4 DNA Ligase (Burlington, ON, CA). The ligated products were transformed into TOP10 *E. coli* competent cells (BioSCI RES, Shanghai, China). Plasmids were extracted by EndoFree Maxi Plasmid Kit (Tiangen, Beijing, China). 293T cells were co-transfected with shRNA BR-V-108 Vector, Helper 1.0 and Helper 2.0 plasmids with Lipofectamine 2000 transfection reagent (Thermo Fisher Scientific, Waltham, MA, USA). Lentiviral vector carrying NDUFC1 gene was successfully constructed.

MGC-803 and SGC-7901 cells were cultured in a 6-cm dish, 40 μl 1×10^8^ TU/ml lentivirus (LV-shCtrl and LV-shNDUFC1) were transfected into MGC-803 and SGC-7901 cells with ENI.S and Polybrene additives. After 72 h culturing, fluorescence and cell infection efficiency were observed and valued by microscopic.

### RNA Extraction and RT-qPCR

Total RNA was extracted from fully lysed shCtrl-MGC-803 and shNDUFC1-SGC-7901 cells using TRIzol reagent (Sigma, St. Louis, MO, USA). The quality of total RNA was evaluated by Nanodrop 2000/2000C spectrophotometer (Thermo Fisher Scientific, Waltham, MA, USA) according to the manufacturer’s instructions. Approximately 2.0 μg total RNA was reverse transcribed using Promega M-MLV Kit (Promega, Heidelberg, Germany). Quantitative real-time PCR (RT-qPCR) was performed with SYBR Green mastermixs Kit (Vazyme, Nangjing, Jiangsu, China) and Biosystems 7500 Sequence Detection system. We used GAPDH as the inner control, and the primers for the PCR reaction were NDUFC1 5’-AGTGCGATCAAAGTTCTACGTG-3’, 5’-AGAAGACAGTGGTGCCCAAG-3’ and human GAPDH 5’-TGACTTCAACAGCGACACCCA-3’. 5’-CACCCTGTTGCTGTAGCCAAA-3’. Reactions were performed in triplicate and the relative quantitative analysis in gene expression data was analyzed by the 2^−ΔΔCt^ method.

### Western Blotting

Lentivirus (LV-shCtrl and LV-shNDUFC1) transfected MGC-803 and SGC-7901 cells were lysed in ice-cold RIPA buffer (Millipore, Temecula, CA, USA). After centrifugation at 12,000*g* for 5 min at 4°C, the protein concentration was detected by BCA Protein Assay Kit (HyClone-Pierce, Logan, UT, USA). Samples (20 μg per lane) were separated by 10% SDS-PAGE (Invitrogen, Carlsbad, CA, USA). All proteins were transferred onto PVDF membranes at 4°C and the PVDF membranes were blocked with TBST solution containing 5% degreased milk at room temperature for 1 h. Then PVDF membranes were incubated with primary antibodies at 4°C overnight. Next, the membranes were incubated with secondary antibody HRP goat anti-rabbit IgG for 2 h at room temperature. The blots were visualized by enhanced chemiluminescence (ECL) (Amersham, Chicago, IL, USA). GAPDH was detected as a loading control in the western blotting. Antibodies used here were shown in **Table S1**.

### MTT Assay

After lentivirus transfection, 100 μl MGC-803 and SGC-7901 cell suspension at a density of 200 cells/μl were seeded into a 96-well plate in triplicate. For coloring, 20 μl 5 mg/mL MTT solution (GenView, El Monte, CA, USA) was added and incubated for 4 h. Approximately 100 μl DMSO solution was added, which was used to dissolve Formazan. The absorbance values at 490 nm were measured by microplate reader (Tecan, Männedorf, Zürich, Switzerland) and the reference wavelength was 570 nm. The cell viability ratio was calculated.

### CCK8 Assay

After SC79 treatment, 100 μl MGC-803 cell suspension with shNDUFC1 was added in a 96-well plate at a cell density 2,000 cell/well. From the second day, 10 μl CCK-8 reagent was added to the well for 2-4 h before the termination of the culture. After 4 h, the 96-well plate was placed on a shaker and oscillated for 2–5 min, and the OD value was detected by the microplate reader at 450 nm.

### Cell Apoptosis and Cycle Assay

After lentivirus transfection, MGC-803 and SGC-7901 cells were inoculated in a 6-well plate with 2 ml per well at a seeding density of 1 × 10^3^ cells/ml in triplicate and further cultured for 5 days. Cells were collected, trypsinized and washed with 4°C ice-cold D-Hanks. After centrifugation (1200×*g*), cells were resuspended with binding buffer, then 5 μl Annexin V-APC (eBioscience, San Diego, CA, USA) was added and used for staining without light. Apoptosis analyses were measured using FACSCalibur (BD Biosciences, San Jose, CA, USA).

The cells were synchronized before cell cycle analysis. Cells were cultured in 2 mM thymidine for 16 h followed by release into medium containing nocodazole (100 ng/ml) for 16 h. The efficiency of synchronization was confirmed by propidium iodide-based cell cycle analysis using flow cytometry. Cells were stained by adding cell staining solution, containing 40 × PI (2 mg/ml), 100 × RNase (10 mg/ml) and 1× PBS. FACSCalibur (BD Biosciences, San Jose, CA, USA) was used to detect cell cycle distribution and the percentages of cells in G1, S, and G2-M phases were analyzed.

### Wound Healing Assay

After lentivirus transfection, MGC-803 and SGC-7901 cells (5 × 10^4^ cells/well) were plated into a 96-well dish in triplicate for culturing. Scratches were made by a 96 wounding replicator (VP scientific, San Diego, CA, USA) across the cell layer with 90% confluence. Photographs were taken by a fluorescence microscope at 0, 8, and 24 h after scratching. Cell migration rate of each group was calculated.

### Transwell Assay

Cell migration was assessed by transwell assay with Corning Transwell Kit (Corning, NT, USA). LV-shCtrl and LV-shNDUFC1 transfected MGC-803 and SGC-7901 cells were incubated in the upper chamber with 100 μl medium without FBS in a 24-well plate (5 × 10^4^ cells/well). Approximately 600 μl medium supplemented with 30% FBS was added into the lower chamber. Cells were incubated for 24 h at 37°C with 5% CO_2_. Finally, lower chamber cells were fixed by 4% formaldehyde and stained by Giemsa and cells from five random fields were selected for observing and the migration ability of cells was analyzed. Experiment was repeated in three wells.

### Human Apoptosis Antibody Array

Human apoptosis signaling pathway was performed using Human Apoptosis Antibody Array (R&D Systems, Minneapolis, MN, USA) following the manufacturer’s instructions. Briefly, lentivirus transfected MGC-803 cells were collected, washed and lysed in ice-cold RIPA buffer (Millipore, Temecula, CA, USA). After centrifugation at 12,000*g* for 5 min at 4°C, the protein concentration was detected by BCA Protein Assay Kit (HyClone-Pierce, Logan, UT, USA). Protein samples (0.5 mg/ml) were incubated with blocked array antibody membrane overnight at 4°C. After washing, 1:100 Detection Antibody Cocktail was added to incubate for 1 h, followed by incubated with HRP linked streptavidin conjugate for 1 h. All spots were visualized by enhanced chemiluminescence (ECL) (Amersham, Chicago, IL, USA) and the signal densities were analyzed with ImageJ software (National Institute of Health, Bethesda, MD, USA).

### Nude Mice Xenograft Tumor Formation

For nude mice xenograft tumor formation, 4 week-old female BALB/c nude mice were purchased from Shanghai Lingchang Experimental Animals Co., Ltd (Shanghai, China) and housed at SPF lab condition. Ten mice were randomized into shCtrl and shNDUFC1 group. SGC-7901 cells were transfected with 4 × 10^6^ lentivirus and were subcutaneously injected into each mouse. Mice’s weight and the tumor sizes were recorded using a caliper. Tumor volume was calculated as L × W^2^ × 0.5 (W means width at the widest point; L means perpendicular width). *In vivo* fluorescence imaging was observed using the IVIS Spectrum Imaging System (Perkin Elmer, Waltham, MA, USA). Finally, mice were killed and tumors were isolated and imaged. All animal studies were approved by the Institutional Animal Care and Use Committee of National Cancer Center/Cancer Hospital, PUMC&CAMS.

For Ki-67 staining, tumor tissues were fixed using formalin and paraffin-embedded. Approximately 5 μm slides were cut and immersed in xylene and 100% ethanol for deparaffinization and rehydration. All slides were blocked with PBS-H_2_O_2_ and were incubated with primary antibody Ki-67 at 4°C overnight. Then slides were incubated with secondary antibody goat anti-rabbit IgG HRP. Finally, all slides were stained by Hematoxylin and Eosin (Baso, Zhuhai, Guangdong, China). Stained slides were examined with a microscopic at 100× and 200× objective lens.

### Statistical Analyses

All data were shown as mean ± standard deviation (SD). The sign test was used to analyze differences in NDUFC1 levels in gastric cancer tissues and normal tissues. Two group comparisons were conducted using a two-tailed Student’s *t*-test. Multiple comparisons were carried out by one-way analysis of variance (ANOVA) test with *post hoc* test by Student–Newman–Keuls test (SNK). The relationship between NDUFC1 expression and tumor characteristics in patients with gastric cancer was analyzed *via* Chi-squared test and Spearman’s rank correlation coefficient analysis. Statistical significance (*P* value) was calculated by SPSS 22.0 (IBM, SPSS, Chicago, IL, USA), and *P* value <0.05 was considered statistically significant. Graphs were made using GraphPad Prism 6.01 (Graphpad Software, La Jolla, CA, USA).

## Results

### Gastric Cancer Was Associated With An Upregulation of NDUFC1

**Figure 1:**
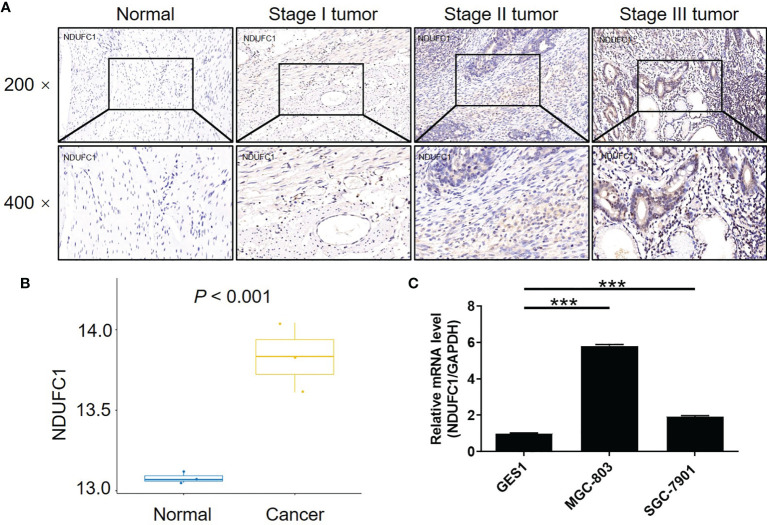
NDUFC1 was upregulated in gastric cancer. **(A)** The expression of NDUFC1 in tumor tissues of gastric cancer was detected by IHC staining and compared with normal tissues, showing that NDUFC1 was upregulated in gastric cancer and associated with tumor stage. **(B)** The NDUFC1 levels were analyzed in gastric cancer tumor and non-tumor tissues from GEO database. **(C)** Background expression of NDUFC1 in normal gastric mucosal cell GES1 and gastric cancer cell lines including MGC-803 and SGC-7901 was detected by qPCR, indicating highly abundant expression of NDUFC1 in gastric cancer cells. The representative images were randomly selected from at least 3 independent experiments. The data was shown as mean ± SD. ***P < 0.001.

To investigate the role of NDUFC1 in the development of gastric cancer, we used immunohistochemical (IHC) analysis to compare the expression of NDUFC1 in tumors collected from patients with gastric cancer and those collected from normal tissues. IHC analysis (*P <*0.05, **Figure 1A** and **Table 1**) showed that NDUFC1 was significantly upregulated in gastric cancer tissues. Further analysis demonstrated that tumors of a more advanced stage expressed elevated levels of NDUFC1 (**Figure 1A**). Additionally, we analyzed NDUFC1 levels in gastric cancer tumor and non-tumor tissues from the GEO database (https://www.ncbi.nlm.nih.gov/geo/query/acc.cgi?acc=GSE49051), suggesting that NDUFC1 was elevated in gastric cancer tissues (**Figure 1B**). Next, we used the Chi-squared test to evaluate the relationship between NDUFC1 expression levels and specific tumor characteristics in gastric cancer patients (**Table 2**). This demonstrated that high expression levels of NDUFC1 were positively correlated with more serious tumor infiltrates, a higher risk of lymphatic metastasis, a higher proportion of positive lymph nodes, and a more advanced tumor stage, which was confirmed by Spearman rank correlation analysis (**Table 3**). We also used qPCR to detect the background expression of NDUFC1 in human normal gastric mucosal cell GES1 and both human gastric cancer cell lines MGC-803 and SGC-7901, showing that the mRNA levels of NDUFC1 in gastric cancer cell lines were higher than that of NDUFC1 in GES1 cells (**Figure 1C**). Collectively, these results indicated that NDUFC1 may play a critical role in the development and progression of gastric cancer.

**Table 1:** Expression patterns of NDUFC1 in gastric cancer tissues and normal tissues revealed in immunohistochemistry analysis.

NDUFC1 expression	Tumor tissue		Normal tissue	
	Cases	Percentage	Cases	Percentage
Low	38	43.2%	87	100%
High	50	56.8%	0	0

P < 0.05.

**Table 2:** Relationship between NDUFC1 expression and tumor characteristics in patients with gastric cancer.

Features	No. of patients	NDUFC1 expression		*P* value
		low	high	
All patients	88	38	50	
Age (years)				0.502
<65	43	17	26	
≥65	45	21	24	
Gender				0.299
Male	67	31	36	
Female	21	7	14	
Tumor infiltrate				0.036
T1	4	4	0	
T2	9	4	5	
T3	56	25	31	
T4	19	5	14	
Lymphatic metastasis (N)				0.004
N0	13	10	3	
N1	12	8	4	
N2	20	6	14	
N3	43	14	29	
Stage				0.007
I	5	4	1	
II	16	10	6	
III	65	24	46	
IV	2	0	1	
Tumor size				0.978
<5 cm	35	15	20	
≥5 cm	47	20	27	
Lymph node positive				0.020
<7	43	24	19	
≥7	45	14	31	
Vessel carcinoma embolus				0.297
No	23	12	11	
Yes	39	15	24	
Nerve tumor infiltrates				0.143
No	32	17	15	
Yes	11	3	8	
Expression of Ki-67				0.821
<55%	39	17	20	
≥55%	39	18	21	
Expression of CD34				0.881
No	12	5	7	
Yes	18	7	11	
Expression of EGFR				0.385
No	66	31	35	
Yes	12	4	8	
Expression of VEGF				0.251
No	20	7	13	
Yes	56	28	28	
Expression of CDX2				0.415
No	9	3	6	
Yes	69	33	36	
Expression of Her2				0.647
No	59	26	33	
Yes	20	10	10	

**Table 3:** Relationship between NDUFC1 expression and tumor characteristics in patients with gastric cancer analyzed by Spearman rank correlation analysis.

Tumor characteristics	index	
Stage	Spearman correlation	0.291
	Significance (two tailed)	0.006
	N	88
Tumor infiltrate	Spearman correlation	0.224
	Significance (two tailed)	0.036
	N	88
Lymph node positive	Spearman correlation	0.249
	Significance (two tailed)	0.019
	N	88
Lymphatic metastasis (N)	Spearman correlation	0.305
	Significance (two tailed)	0.004
	N	88

### The Creation of Cell Models For Gastric Cancer by NDUFC1 Knockdown

**Figure 2:**
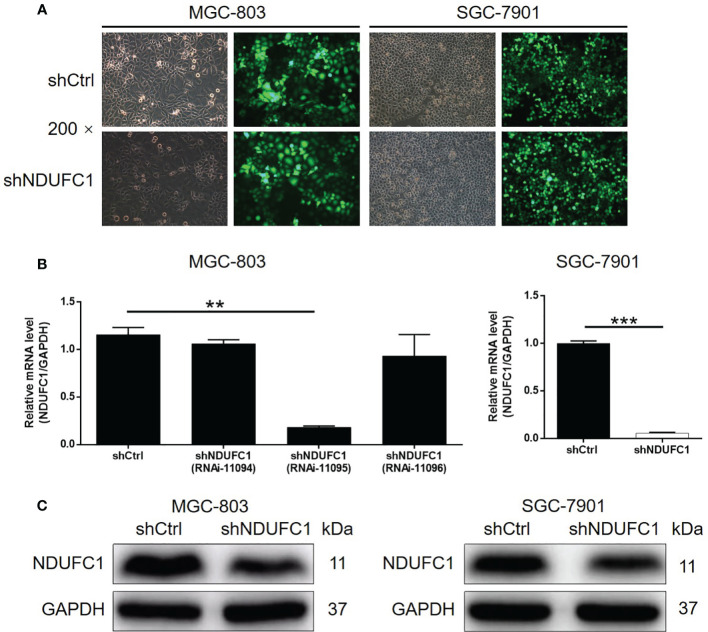
Successful construction of gastric cancer cell models with NDUFC1 knockdown. **(A)** Fluorescence imaging was performed to evaluate the efficiency of shNDUFC1 lentivirus transfection in MGC-803 and SGC-7901 cells. **(B)** qPCR was utilized to detect the knockdown efficiency of NDUFC1 in MGC-803 and SGC-7901 cells. **(C)** The successful knockdown of NDUFC1 in MGC-803 and SGC-7901 cells was further verified by western blotting. GAPDH was detected as a loading control in the western blotting. The representative images were randomly selected from at least 3 independent experiments. The data was shown as mean ± SD. **P < 0.01, ***P < 0.001.

Our previous results suggested that NDUFC1 may act to promote gastric cancer. Next, we constructed NDUFC1 knockdown cell lines using MGC-803 and SGC-7901 cells. Specifically, we transfected each cell line with a lentivirus expressing shRNAs designed to silence NDUFC1. The efficiencies of transfection were evaluated by monitoring green fluorescence from the GFP expressed by the lentivirus vector; an efficiency in excess of 80% was considered to represent successful transfection (**Figure 2A**). Next, we used qPCR to measure the knockdown efficiencies of NDUFC1 in both MGC-803 and SGC-7901 cell lines (**Figure 2B**). Of the three RNAi designs created to silence NDUFC1, RNAi-11095 exhibited the most powerful knockdown of NDUFC1 (84.3%, *P <*0.01) in MGC-803 cells; consequently, this RNAi was used in all subsequent experiments. The knockdown of NDUFC1 in SGC-7901 cells was 94.4% (*P <*0.001), thus indicating the successful establishment of cell models in which NDUFC1 had been knocked down (**Figure 2B**). We also used western blotting to conform that NDUFC1 had been depleted inMGC-803 and SGC-7901 cells (**Figure 2C** and **Figure S1A**). Collectively, these results indicated that we had successfully established cell models of gastric cancer in which NDUFC1 had been knocked down.

### The Knockdown of NDUFC1 Inhibited the Proliferation of Gastric Cancer Cells and Promoted Apoptosis and Cell Cycle Arrest

**Figure 3:**
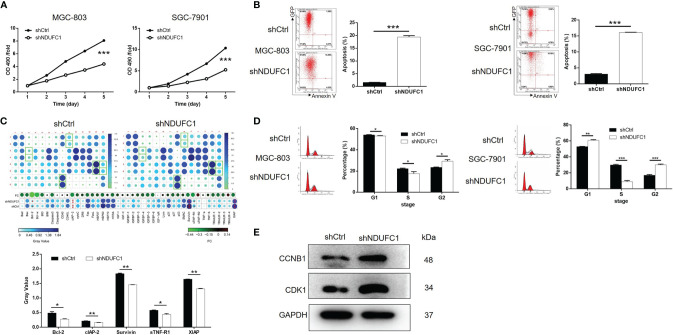
NDUFC1 knockdown inhibited cell proliferation and promoted cell apoptosis and cell cycle arrest in gastric cancer cells. **(A)** MTT assay showed that knockdown of NDUFC1 significantly inhibited cell proliferation of MGC-803 and SGC-7901 cells. The “OD490/fold” meant the multiple of each OD490 on day 1-5 relative to the OD490 on day 1, which represented the multiple of proliferation on each day. **(B)** The results of flow cytometry demonstrated that knockdown of NDUFC1 obviously promoted cell apoptosis of MGC-803 and SGC-7901 cells. **(C)** Human Apoptosis Antibody Array was performed in MGC-803 cells to identify the differential expression of apoptosis-related proteins between shCtrl and shNDUFC1 groups and showed the downregulation of Bcl-2, clAP-2, Survivin, sTNF-R1 and XIAP in shNDUFC1 group. **(D)** The detection of MGC-803 and SGC-7901 cell cycle *via* flow cytometry indicated that knockdown of NDUFC1 significantly promoted the arrest of cell cycle in G2/M phase. The cells were synchronized using 2mM thymidine and nocodazole (100 ng/ml) before cell cycle analysis. **(E)** Western blotting assay indicated that the expression of cyclin B1 and Cdk1 increased in response to NDUFC1 knockdown. The representative images were randomly selected from at least 3 independent experiments. The data was shown as mean ± SD. *P < 0.05, **P < 0.01, ***P < 0.001.

To investigate how NDUFC1 influences the development of gastric cancer, we carried out a range of experiments to investigate cell proliferation, cell apoptosis, and cell cycle in MGC-803 and SGC-7901 cells with or without NDUFC1 knockdown. The MTT assay was used to detect cell proliferation; our analysis showed that the knockdown of NDUFC1 significantly inhibited the growth of both MGC-803 and SGC-7901 cells (*P <*0.001, **Figure 3A**). The knockdown of NDUFC1 also led to a significant elevation (by five-fold) of apoptosis in MGC-803 and SGC-7901 cells (*P <*0.001, **Figure 3B**). To identify the potential mechanisms by which the knockdown of NDUFC1 regulates apoptosis in these cells, we performed a human apoptosis antibody array to identify differentially expressed proteins related to apoptosis in the shNDUFC1 group of MGC-803 cells and compared this to the shCtrl group of cells. As shown in **Figure 3C**, the knockdown of NDUFC1 induced the significant downregulation of Bcl-2, clAP-2, Survivin, sTNF-R1, and XIAP (*P <*0.05, **Figure 3C**), implying that NDUFC1 knockdown might regulate cell apoptosis by a manner of moderating these proteins. Furthermore, cell cycle analysis revealed that NDUFC1 knockdown caused MGC-803 and SGC-7901 cell cycle arrest at G2/M phase (*P <*0.05, **Figure 3D**). As we know, cyclin B1/CDK1 complex, the critical target of G2/M checkpoint, plays critical roles in mitosis (17, 18). Western blotting indicated that the expression of cyclin B1 and Cdk1 was increased in response to NDUFC1 knockdown (**Figure 3E** and **Figure S1B**). Herein, NDUFC1 knockdown induced gastric cancer cells G2/M phase arrest through regulation of cyclin B1/Cdk1 complex. Collectively, these results showed that NDUFC1 played a role in the development of gastric cancer.

### The Knockdown of NDUFC1 Inhibited Cell Migration and the Activity of the PI3K/AKT Signaling Pathway

**Figure 4:**
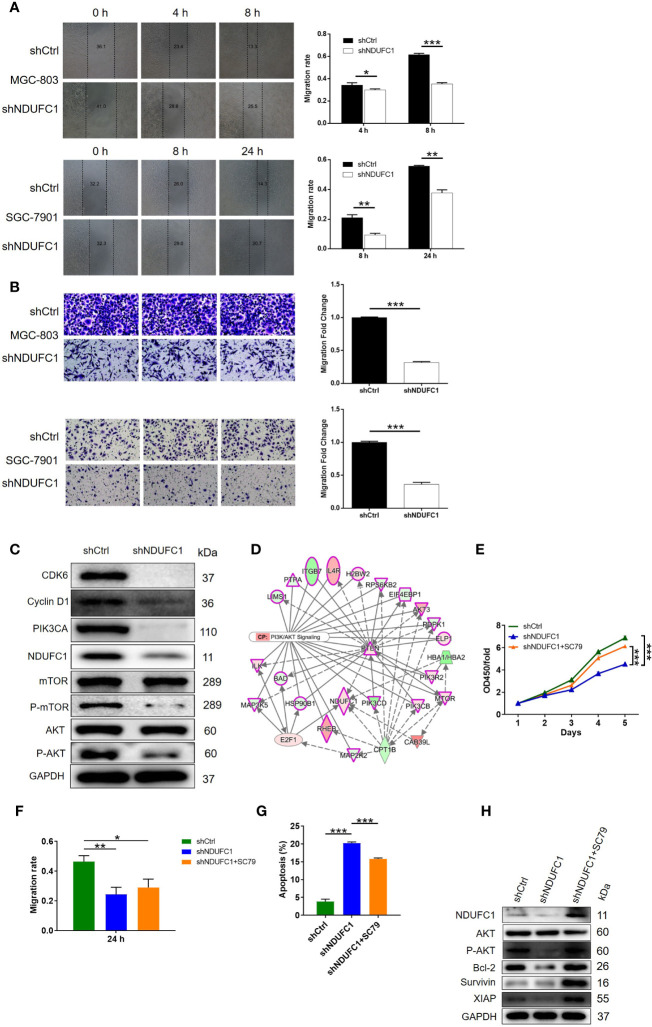
NDUFC1 knockdown inhibited cell migration and the activity of the PI3K/AKT signaling pathway. **(A)** Wound-healing assay showed that cell migration of MGC-803 and SGC-7901 cells was significantly suppressed by NDUFC1 knockdown. **(B)** The results of Transwell assay clarified that knockdown of NDUFC1 could clearly inhibited cell migration of MGC-803 and SGC-7901 cells. **(C)** The results of western blotting showed the downregulation of several cancer-associated factors such as CDK6, Cyclin D1,PIK3CA, P-mTOR and P-AKT as well as NDUFC1 in shNDUFC1 group. **(D)** An interaction network between NDUFC1 and PI3K-Akt signaling pathway was constructed based on Ingenuity pathway analysis (IPA). **(E)** CCK8 assay showed the alterations of MGC-803 cell proliferation from shCtrl, shNDUFC1 and shNDUFC1 + SC79 groups after treating with or without AKT signaling pathway agonist: SC79 (10 μM). **(F)** Wound-healing assay was performed to assess cell migration of MGC-803 cells from shCtrl, shNDUFC1 and shNDUFC1 + SC79 groups. **(G)** The results of flow cytometry demonstrated that SC79 treatment obviously reversed promoted MGC-803 cell apoptosis induced by NDUFC1 depletion. **(H)** Western blotting showed protein expression of AKT, p-AKT, Bcl-2, Survivin and XIAP in shCtrl, shNDUFC1 and shNDUFC1 + SC79 in MGC-803 cells treated with or without AKT signaling pathway agonist: SC79 (10 μM). GAPDH was detected as a loading control in the western blotting. The representative images were randomly selected from at least 3 independent experiments. The data was shown as mean ± SD. *P < 0.05, **P < 0.01, ***P < 0.001.

Next, we used wound-healing and transwell assays to investigate the effects of NDUFC1 knockdown on cell migration ability and tumor metastasis. We found that the knockdown of NDUFC1 led to a significant suppression of migration in MGC-803 and SGC-7901 cells; this was confirmed by both wound-healing and transwell assays (*P <*0.001, **Figures 4A, B**). After confirming that NDUFC1 played a role in the development and metastasis of gastric cancer, we next turned our attention to mechanisms that might be responsible for these effects. As shown in **Figure 4C**, we used western blotting to investigate the expression of several factors that are known to play significant roles in human cancers, including CDK6, Cyclin D1, PIK3CA, P-AKT, and P-mTOR. Our analysis showed that the levels of all of these factors were downregulated in the shNDUFC1 group of MGC-803 cells (**Figure 4C** and **Figure S1C**). On the other hand, we found that NDUFC1 was related to PI3K-Akt signaling pathway through an analysis of interaction network based on Ingenuity pathway analysis (IPA) (**Figure 4D**). It is well-known that PI3K/AKT signaling pathway is one of the most important signaling pathways involved in normal cellular processes. Its aberrant activation modulates autophagy, epithelial–mesenchymal transition, apoptosis, chemoresistance, and metastasis in many human cancers including gastric cancer. More importantly, PI3K-Akt signaling pathway is a potential target by which multiple genes promotes gastric cancer cell proliferation and migration (19, 20). We speculated that NDUFC1 might regulate gastric cancer *via* PI3K-Akt signaling pathway as well. Therefore, we treated MGC-803 cells with or without AKT signaling pathway agonist: SC79 (10 μM). We found that SC79 rescued the inhibitory effects on cell proliferation and migration, while reversing the promoted effects on cell apoptosis caused by NDUFC1 knockdown (**Figures 4E**–**G**). More importantly, NDUFC1 knockdown inhibited the expression level of p-AKT, NDUFC1, Bcl-2, Survivin, and XIAP relative to shCtrl group. Whereas, compared with NDUFC1 knockdown group, the expression of these proteins was up-regulated in shNDUFC1+SC79 group (**Figure 4H** and **Figure S1D**). Our suspicion was that NDUFC1 knockdown blocked gastric cancer’s progression *via* PI3K/AKT signaling pathway.

### The Knockdown of NDUFC1 Inhibited Gastric Cancer Tumor Growth *In Vivo*

**Figure 5:**
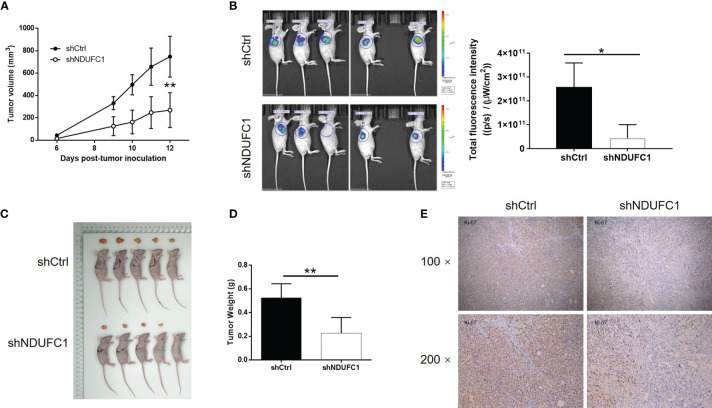
NDUFC1 knockdown inhibited tumor growth of gastric cancer *in vivo.* **(A)** In xenograft tumor models, the volume of tumors was measured and showed that depleting NDUFC1 obviously slowed down growth of tumor. **(B)** The fluorescence intensity obtained by *in vivo* imaging showed apparently smaller tumors in shNDUFC1 group. **(C)** The photos of the removed tumors were obtained after sacrificing the animals. **(D)** The weight of the tumors was measured, which showed that tumors in shNDUFC1 group were lighter. **(E)** The IHC analysis of Ki-67 expression in tumors showed obvious higher levels in shCtrl group. The representative images were randomly selected from at least 3 independent experiments. The data was shown as mean ± SD. *P < 0.05, **P < 0.01.

Next, we attempted to verify the roles of NDUFC1 in gastric cancer *in vivo*. To do this, we subcutaneously injected MGC-803 cells with or without NDUFC1 knockdown into mice in order to establish a mouse xenograft models. Animals were then maintained and observed regularly. At the end of the experimental period, we analyzed tumor growth and calculated the volume of tumor based on their size. We found that the knockdown of NDUFC1 significantly slowed down the rate of tumor growth *in vivo* (*P <*0.01, **Figure 5A**). We also injected D-Luciferin into mice so that we could monitor the suppression of tumor growth in response to NDUFC1 knockdown by fluorescence imaging (*P <*0.05, **Figure 5B**). Animals were sacrificed at the end of the experiment. At this point, we acquired photographs of the tumors and weighed each tumor individually. Analysis showed that the tumors in the shNDUFC1 groups were significantly smaller than those in the other groups (*P <*0.01, **Figures 5C, D**). Furthermore, we also detected Ki-67 levels in each of the removed tumors. Ki-67, a proliferation marker, is considered to represent an excellent index of tumor growth (21, 22) and was downregulated in the shNDUFC1 group (*P <*0.05, **Figure 5E**). Collectively, these results suggested that the knockdown of NDUFC1 could restrain tumor growth *in vivo*.

## Discussion

Gastric cancer is one of the most common malignant tumors in the digestive system. It possesses the fifth highest morbidity worldwide and is the third leading cause of cancer-related deaths (1). The progress and metastasis of gastric cancer is a complex process involving multiple genes and pathways, the molecular mechanism of which has not been fully elucidated till now (23). It is of great significance to find valuable molecular therapeutic targets in the intricate signaling pathways for the targeted treatment of gastric cancer (24). Previous studies have indicated the involvement of various genes or signaling pathways in the development of gastric cancer (25, 26). For example, accumulating evidence showed that overexpression of Human epidermal growth factor receptor-2 (HER2) could promote proliferation, migration and infiltration of gastric cancer cells, which has also been used in the targeted therapy of gastric cancer (27–29). Besides, c-Met was also reported to be highly expressed in gastric cancer and associated with infiltration, metastasis and poor prognosis of gastric cancer (30–32). Moreover, Wnt/β-catenin signaling pathway was found to be able to regulate differentiation, invasion, and metastasis of gastric cancer through its downstream such as HMGA1 or SOX17 (33–35). MAPK, a canonical cancer-related signaling pathway, was also proposed to have critical regulation effects in the development of gastric cancer (36, 37). Collectively, the exploration of abnormally expressed genes in gastric cancer and identification of them as promising therapeutic targets in treatment of gastric cancer are of great significance for patients with gastric cancer.

NADH: ubiquinone oxidoreductase (complex I) is the first, and also largest and most complex electron translocating enzyme in the electron transport chain in mitochondria. As a multi-subunit complex, various subunits of NADH: ubiquinone oxidoreductase (complex I) have been identified, isolated and investigated (15). For example, nuclear DNA-encoded NADH: ubiquinone oxidoreductase subunit A9 (NDUFA9) was found to be relatively upregulated in human colorectal cancer cell line SW620 and may exert a promotion effects on invasion of CRC (38). Elkholi reported that NADH: ubiquinone oxidoreductase 75 kDa Fe–S protein 1 (NDUFS1), another subunit of NADH: ubiquinone oxidoreductase (complex I), mediated the regulation of mitochondrial respiration, oxidative stress, and mitochondrial pathway of apoptosis by MDM2 (39). Liu reported that mitochondrial complex I subunit NADH dehydrogenase (ubiquinone) Fe–S protein 2 (NDUFS2) played an important role in the metabolism and invasion of lung cancer, which may be regulated by S100 calcium-binding protein A4 (S100A4) (40). Moreover, the amplification of NADH:ubiquinone oxidoreductase subunit C2 (NDUFC2) was identified and found to be associated with worse prognosis of ER negative breast cancer (41). Despite of these results, the role of NDUFC1 in cancer, including gastric cancer was rarely seen and remains unknown.

In this study, we found that NDUFC1 was significantly upregulated in gastric cancer tissues compared with normal tissues. Moreover, further statistical analysis revealed that high NDUFC1 expression was associated with more serious tumor infiltration, more advanced tumor stage and higher risk of lymphatic metastasis. Subsequently, the investigation of biological behaviors of gastric cancer cell lines MGC-803 and SGC-7901, with or without knockdown of NDUFC1, showed that knockdown of NDUFC1 significantly inhibited cell proliferation and cell migration, while promoting cell apoptosis and arrest of cell cycle in G2/M phase. Furthermore, *in vivo* studies using mice xenograft model constructed by MGC-803 cells also demonstrated the suppressed growth of tumors and the restrained expression of Ki-67 in tumors, which was in consistent with the *in vitro* experiments. All the results provided clear evidence that NDUFC1 knockdown could obviously alleviate the development of gastric cancer and may be a therapeutic target for the treatment of gastric cancer.

Using Human apoptosis antibody array, we found that NDUFC1 knockdown may promote gastric cancer cell apoptosis through downregulating Bcl-2, clAP-2, Survivin, sTNF-R1, and XIAP. Further detection of the expression levels of CDK6 and Cyclin D1, which plays critical roles in cell cycle and is well known to be involved in the development of various types of human cancers including gastric cancer (42, 43), showed downregulated expression induced by NDUFC1 knockdown. Otherwise, the downregulation of PIK3CA, a well-known oncogene (44), was also observed in MGC-803 cells with NDUFC1 knockdown and supposed to participate in the NDUFC1 induced regulation of gastric cancer. It must also be mentioned that PI3K/AKT is an important participant in the regulation of cell growth, proliferation, survival and metabolism (19, 45). We found that the phosphorylation levels of AKT and mTOR were decreased upon NDUFC1 knockdown, and that the addition of AKT pathway agonist SC79 could rescue the inhibitory effects of NDUFC1 knockdown on cell proliferation and migration, while reversing cell apoptosis. What’s more striking was that P-AKT, Bcl-2, Survivin, and XIAP were augmented in NDUFC1-deficient cells upon SC79 treatment. From these, we speculated that NDUFC1 downregulation might suppress gastric cancer’s progression *via* PI3K/AKT signaling pathway.

In conclusion, our studies identified NDUFC1 as a tumor promotor of gastric cancer, which was upregulated in gastric cancer and thus promoting gastric cancer through regulation of cell proliferation, cell apoptosis, cell cycle and cell migration. Therefore, NDUFC1 may be considered as a novel therapeutic target and its knockdown may serve as a promising strategy of gastric cancer treatment.

## Data Availability Statement

The original contributions presented in the study are included in the article/**Supplementary Material**. Further inquiries can be directed to the corresponding author.

## Ethics Statement

All studies were approved by the Ethics Committee of National Cancer Center/Cancer Hospital, PUMC & CAMS. The patients/participants provided their written informed consent to participate in this study. All animal studies were approved by the Institutional Animal Care and Use Committee of National Cancer Center/Cancer Hospital, PUMC & CAMS.

## Author Contributions

YS designed this program and XC and HJ operated the cell and animal experiments. JX and LW conducted the data collection and analysis. LX produced the manuscript which was checked by YS. All authors contributed to the article and approved the submitted version.

## Funding

We acknowledge the Hainan Provincial Natural Science Foundation of China (No. 820QN404).

## Conflict of Interest

The authors declare that the research was conducted in the absence of any commercial or financial relationships that could be construed as a potential conflict of interest.

## Publisher’s Note

All claims expressed in this article are solely those of the authors and do not necessarily represent those of their affiliated organizations, or those of the publisher, the editors and the reviewers. Any product that may be evaluated in this article, or claim that may be made by its manufacturer, is not guaranteed or endorsed by the publisher.

## References

[1] Bray F Ferlay J Soerjomataram I Siegel RL Torre LA Jemal A. Global Cancer Statistics 2018: GLOBOCAN Estimates of Incidence and Mortality Worldwide for 36 Cancers in 185 Countries. CA Cancer J Clin (2018) 68:394–424. doi: 10.3322/caac.21492 30207593

[2] Jayavelu ND Bar NS. Metabolomic Studies of Human Gastric Cancer: Review. World J Gastroenterol (2014) 20:8092–101. doi: 10.3748/wjg.v20.i25.8092 PMC4081680 25009381

[3] Hudler P. Outlook on Epigenetic Therapeutic Approaches for Treatment of Gastric Cancer. Curr Cancer Drug Targets (2018) 18:65–88. doi: 10.2174/1568009617666170203163745 28176656

[4] Fock KM. Review Article: The Epidemiology and Prevention of Gastric Cancer. Aliment Pharmacol Ther (2014) 40:250–60. doi: 10.1111/apt.12814 24912650

[5] Maeda O Ando Y. Recent Progress of Chemotherapy and Biomarkers for Gastroesophageal Cancer. World J Gastrointest Oncol (2019) 11:518–26. doi: 10.4251/wjgo.v11.i7.518 PMC6657220 31367271

[6] Dong H Liu H Zhou W Zhang F Li C Chen J. GLI1 Activation by Non-Classical Pathway Integrin α(V)β(3)/ERK1/2 Maintains Stem Cell-Like Phenotype of Multicellular Aggregates in Gastric Cancer Peritoneal Metastasis. Cell Death Dis (2019) 10:574. doi: 10.1038/s41419-019-1776-x 31366904 PMC6668446

[7] Niu G Yang Y Ren J Song T Hu Z Chen L. Overexpression of CPXM2 Predicts an Unfavorable Prognosis and Promotes the Proliferation and Migration of Gastric Cancer. Oncol Rep (2019) 42:1283–94. doi: 10.3892/or.2019.7254 PMC6718098 31364750

[8] Jo MJ Jeong S Yun HK Kim DY Kim BR Kim JL. Genipin Induces Mitochondrial Dysfunction and Apoptosis *via* Downregulation of Stat3/mcl-1 Pathway in Gastric Cancer. BMC Cancer (2019) 19:739. doi: 10.1186/s12885-019-5957-x 31351462 PMC6661087

[9] Park JE Jin MH Hur M Nam AR Bang JH Won J. GC1118, a Novel Anti-EGFR Antibody, has Potent KRAS Mutation-Independent Antitumor Activity Compared With Cetuximab in Gastric Cancer. Gastric Cancer (2019) 22:932–40. doi: 10.1007/s10120-019-00943-x 30815759

[10] Russo A Li P Strong VE. Differences in the Multimodal Treatment of Gastric Cancer: East Versus West. J Surg Oncol (2017) 115:603–14. doi: 10.1002/jso.24517 28181265

[11] Sitarz R Skierucha M Mielko J Offerhaus GJA Maciejewski R Polkowski WP. Gastric Cancer: Epidemiology, Prevention, Classification, and Treatment. Cancer Manag Res (2018) 10:239–48. doi: 10.2147/CMAR.S149619 PMC5808709 29445300

[12] Weiss H Friedrich T Hofhaus G Preis D. The Respiratory-Chain NADH Dehydrogenase (Complex I) of Mitochondria. Eur J Biochem (1991) 197:563–76. doi: 10.1111/j.1432-1033.1991.tb15945.x 2029890

[13] Chen YR Chen CL Zhang L Green-Church KB Zweier JL. Superoxide Generation From Mitochondrial NADH Dehydrogenase Induces Self-Inactivation With Specific Protein Radical Formation. J Biol Chem (2005) 280:37339–48. doi: 10.1074/jbc.M503936200 16150735

[14] McDonald AE Gospodaryov DV. Alternative NAD(P)H Dehydrogenase and Alternative Oxidase: Proposed Physiological Roles in Animals. Mitochondrion (2019) 45:7–17. doi: 10.1016/j.mito.2018.01.009 29421444

[15] Ton C Hwang DM Dempsey AA Liew CC. Identification and Primary Structure of Five Human NADH-Ubiquinone Oxidoreductase Subunits. Biochem Biophys Res Commun (1997) 241:589–94. doi: 10.1006/bbrc.1997.7707 9425316

[16] Abu Dawud R Schreiber K Schomburg D Adjaye J. Human Embryonic Stem Cells and Embryonal Carcinoma Cells Have Overlapping and Distinct Metabolic Signatures. PloS One (2012) 7:e39896. doi: 10.1371/journal.pone.0039896 22768158 PMC3387229

[17] Castedo M Perfettini JL Roumier T Andreau K Medema R Kroemer G. Cell Death by Mitotic Catastrophe: A Molecular Definition. Oncogene (2004) 23:2825–37. doi: 10.1038/sj.onc.1207528 15077146

[18] Castedo M Perfettini JL Roumier T Valent A Raslova H Yakushijin K. Mitotic Catastrophe Constitutes a Special Case of Apoptosis Whose Suppression Entails Aneuploidy. Oncogene (2004) 23:4362–70. doi: 10.1038/sj.onc.1207572 15048075

[19] Fattahi S Amjadi-Moheb F Tabaripour R Ashrafi GH Akhavan-Niaki H. PI3K/AKT/mTOR Signaling in Gastric Cancer: Epigenetics and Beyond. Life Sci (2020) 262:118513. doi: 10.1016/j.lfs.2020.118513 33011222

[20] Ao R Guan L Wang Y Wang JN. Silencing of COL1A2, COL6A3, and THBS2 Inhibits Gastric Cancer Cell Proliferation, Migration, and Invasion While Promoting Apoptosis Through the PI3k-Akt Signaling Pathway. J Cell Biochem (2018) 119:4420–34. doi: 10.1002/jcb.26524 29143985

[21] Graefe C Eichhorn L Wurst P Kleiner J Heine A Panetas I. Optimized Ki-67 Staining in Murine Cells: A Tool to Determine Cell Proliferation. Mol Biol Rep (2019) 46:4631–43. doi: 10.1007/s11033-019-04851-2 31093875

[22] Menon SS Guruvayoorappan C Sakthivel KM Rasmi RR. Ki-67 Protein as a Tumour Proliferation Marker. Clin Chim Acta (2019) 491:39–45. doi: 10.1016/j.cca.2019.01.011 30653951

[23] Karimi P Islami F Anandasabapathy S Freedman ND Kamangar F. Gastric Cancer: Descriptive Epidemiology, Risk Factors, Screening, and Prevention. Cancer Epidemiol Biomarkers Prev (2014) 23:700–13. doi: 10.1158/1055-9965.EPI-13-1057 PMC4019373 24618998

[24] Yuan DD Zhu ZX Zhang X Liu J. Targeted Therapy for Gastric Cancer: Current Status and Future Directions (Review). Oncol Rep (2016) 35:1245–54. doi: 10.3892/or.2015.4528 26718131

[25] Li C Li X Gao S Li C Ma L. MicroRNA-133a Inhibits Proliferation of Gastric Cancer Cells by Downregulating ERBB2 Expression. Oncol Res (2017) 25:1169–76. doi: 10.3727/096504017X14847395834985 PMC7840978 28109082

[26] Lee SY Oh SC. Changing Strategies for Target Therapy in Gastric Cancer. World J Gastroenterol (2016) 22:1179–89. doi: 10.3748/wjg.v22.i3.1179 PMC4716029 26811656

[27] Boku N. HER2-Positive Gastric Cancer. Gastric Cancer (2014) 17:1–12. doi: 10.1007/s10120-013-0252-z 23563986 PMC3889288

[28] Takegawa N Nonagase Y Yonesaka K Sakai K Maenishi O Ogitani Y. DS-8201a, a New HER2-Targeting Antibody-Drug Conjugate Incorporating a Novel DNA Topoisomerase I Inhibitor, Overcomes HER2-Positive Gastric Cancer T-DM1 Resistance. Int J Cancer (2017) 141:1682–9. doi: 10.1002/ijc.30870 28677116

[29] Gao Z Song C Li G Lin H Lian X Zhang N. Pyrotinib Treatment on HER2-Positive Gastric Cancer Cells Promotes the Released Exosomes to Enhance Endothelial Cell Progression, Which Can Be Counteracted by Apatinib. Onco Targets Ther (2019) 12:2777–87. doi: 10.2147/OTT.S194768 PMC6489591 31114227

[30] Zhao R Zhang T Xi W Sun X Zhou L Guo Y. Human Chorionic Gonadotropin Promotes Cell Proliferation Through the Activation of C-Met in Gastric Cancer Cells. Oncol Lett (2018) 16:4271–8. doi: 10.3892/ol.2018.9215 PMC6126336 30197669

[31] Anestis A Zoi I Karamouzis MV. Current Advances of Targeting HGF/c-Met Pathway in Gastric Cancer. Ann Transl Med (2018) 6:247. doi: 10.21037/atm.2018.04.42 30069449 PMC6046293

[32] Kim HJ Kang SK Kwon WS Kim TS Jeong I Jeung HC. Forty-Nine Gastric Cancer Cell Lines With Integrative Genomic Profiling for Development of C-MET Inhibitor. Int J Cancer (2018) 143:151–9. doi: 10.1002/ijc.31304 29435981

[33] Zhang Z Yu W Zheng M Liao X Wang J Yang D. Pin1 Inhibition Potently Suppresses Gastric Cancer Growth and Blocks PI3K/AKT and Wnt/β-Catenin Oncogenic Pathways. Mol Carcinog (2019) 58:1450–64. doi: 10.1002/mc.23027 PMC6753380 31026381

[34] Tang L Wen JB Wen P Li X Gong M Li Q. Long Non-Coding RNA LINC01314 Represses Cell Migration, Invasion, and Angiogenesis in Gastric Cancer *via* the Wnt/β-Catenin Signaling Pathway by Down-Regulating KLK4. Cancer Cell Int (2019) 19:94. doi: 10.1186/s12935-019-0799-9 31007611 PMC6458728

[35] Akaboshi S Watanabe S Hino Y Sekita Y Xi Y Araki K. HMGA1 Is Induced by Wnt/beta-Catenin Pathway and Maintains Cell Proliferation in Gastric Cancer. Am J Pathol (2009) 175:1675–85. doi: 10.2353/ajpath.2009.090069 PMC2751563 19729480

[36] Han L Xiong L Wang C Shi Y Song Q Sun G. MicroRNA-128 Contributes to the Progression of Gastric Carcinoma Through GAREM-Mediated MAPK Signaling Activation. Biochem Biophys Res Commun (2018) 504:295–301. doi: 10.1016/j.bbrc.2018.08.177 30177387

[37] Yin K Shang M Dang S Wang L Xia Y Cui L. Netrin−1 Induces the Proliferation of Gastric Cancer Cells *via* the ERK/MAPK Signaling Pathway and FAK Activation. Oncol Rep (2018) 40:2325–33. doi: 10.3892/or.2018.6614 30106432

[38] Lin CS Liu LT Ou LH Pan SC Lin CI Wei YH. Role of Mitochondrial Function in the Invasiveness of Human Colon Cancer Cells. Oncol Rep (2018) 39:316–30. doi: 10.3892/or.2017.6087 29138850

[39] Elkholi R Abraham-Enachescu I Trotta AP Rubio-Patiño C Mohammed JN Luna-Vargas MPA. MDM2 Integrates Cellular Respiration and Apoptotic Signaling Through NDUFS1 and the Mitochondrial Network. Mol Cell (2019) 74:452–65.e457. doi: 10.1016/j.molcel.2019.02.012 30879903 PMC6499641

[40] Liu L Qi L Knifley T Piecoro DW Rychahou P Liu J. S100A4 Alters Metabolism and Promotes Invasion of Lung Cancer Cells by Up-Regulating Mitochondrial Complex I Protein NDUFS2. J Biol Chem (2019) 294:7516–27. doi: 10.1074/jbc.RA118.004365 PMC6509482 30885944

[41] Chin SF Teschendorff AE Marioni JC Wang Y Barbosa-Morais NL Thorne NP. High-Resolution aCGH and Expression Profiling Identifies a Novel Genomic Subtype of ER Negative Breast Cancer. Genome Biol (2007) 8:R215. doi: 10.1186/gb-2007-8-10-r215 17925008 PMC2246289

[42] Deng M Zeng C Lu X He X Zhang R Qiu Q. miR-218 Suppresses Gastric Cancer Cell Cycle Progression Through the CDK6/Cyclin D1/E2F1 Axis in a Feedback Loop. Cancer Lett (2017) 403:175–85. doi: 10.1016/j.canlet.2017.06.006 28634044

[43] Ooi A Oyama T Nakamura R Tajiri R Ikeda H Fushida S. Gene Amplification of CCNE1, CCND1, and CDK6 in Gastric Cancers Detected by Multiplex Ligation-Dependent Probe Amplification and Fluorescence *In Situ* Hybridization. Hum Pathol (2017) 61:58–67. doi: 10.1016/j.humpath.2016.10.025 27864121

[44] Arafeh R Samuels Y. PIK3CA in Cancer: The Past 30 Years. Semin Cancer Biol (2019) 59:36–49. doi: 10.1016/j.semcancer.2019.02.002 30742905

[45] Martini M De Santis MC Braccini L Gulluni F Hirsch E. PI3K/AKT Signaling Pathway and Cancer: An Updated Review. Ann Med (2014) 46:372–83. doi: 10.3109/07853890.2014.912836 24897931

