# Supplementary material for: NDUFC1 Is Upregulated in Gastric Cancer and Regulates Cell Proliferation, Apoptosis, Cycle and Migration
Source: Front Oncol. 2021 Dec 13;11:709044. doi: 10.3389/fonc.2021.709044 (PMC8710466; doi:10.3389/fonc.2021.709044)
Supplement: Supplementary file 1 [file DataSheet_1.pdf]

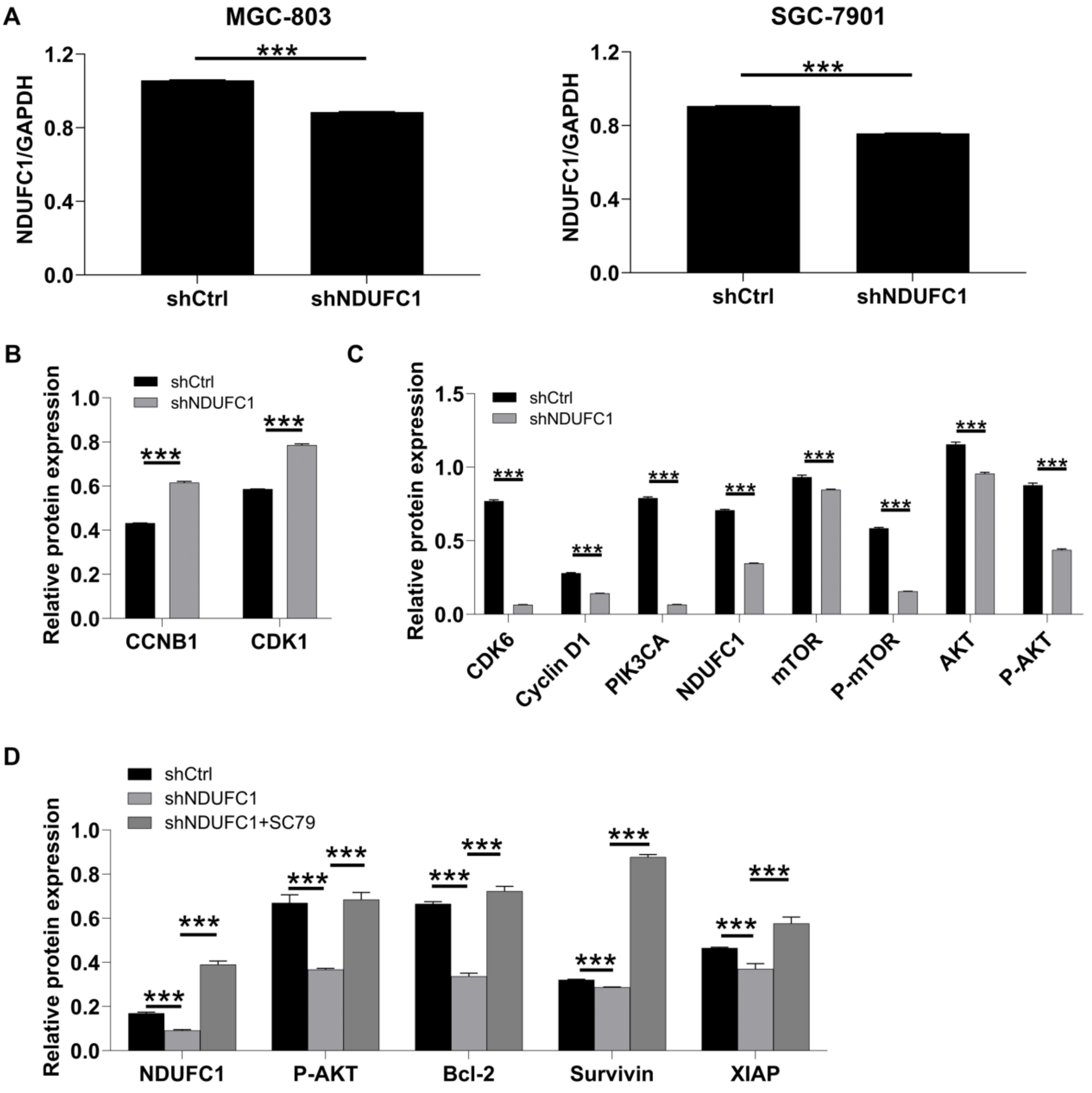

Figure S1. (A) The NDUFC1 protein levels were quantified in MGC-803 and SGC-7901 cells after lentivirus transfection. (B) The protein levels of CCNB1 and CDK1 were quantified in response to NDUFC1 depletion. (C) The protein levels of CDK6, Cyclin D1, PIK3CA, NDUFC1, mTOR, P-mTOR, AKT and P-AKT were quantified in response to NDUFC1 depletion. (D) The protein levels of NDUFC1, P-AKT, Bcl-2, Survivin and XIAP were quantified in response to NDUFC1 depletion and SC79 treatment.

Table S1. Antibodies used in western blotting and IHC.

Primary antibodies	Dilution in WB	Source species	Company	Catalog No.
NDUFC1	1:1000	Rabbit	Invitrogen	PA5-68240
mTOR	1:3000	Mouse	Proteintech	66888-1-Ig
p-mTOR	1:5000	Mouse	Proteintech	67778-1-Ig
AKT	1:1000	Rabbit	CST	4691S
p-AKT	1:1000	Rabbit	Bioss	bs-5193R
CCNB1	1:3000	Rabbit	Proteintech	55004-1-AP
CDK1	1:1000	Mouse	Santa Cruz	sc-54
CDK6	1:1000	Rabbit	abcam	ab151247
Cyclin D1	1:2000	Rabbit	CST	2978
PIK3CA	1:1000	Rabbit	abcam	ab40776
Bcl-2	1:2000	Rabbit	Abcam	ab182858
Survivin	1:2000	Rabbit	Proteintech	10508-1-AP
XIAP	1:1000	Mouse	Abcam	ab28151
GAPDH	1:3000	Rabbit	Bioworld	AP0063
GAPDH	1:3000	Mouse	Proteintech	60004-1-lg
Secondary antibody	Dilution		Company	Catalog No.
Goat Anti-Rabbit	1:3000		Beyotime	A0208
Goat Anti- Mouse	1:3000		Beyotime	A0216

Primary antibodies	Dilution in IHC	Source species	Company	Catalog No.
NDUFC1	1:50	Rabbit	abcam	ab122869
Ki67	1:200	Rabbit	abcam	Ab16667
Secondary antibody	Dilution		Company	Catalog No.
Goat Anti-Rabbit IgG H&L (HRP)	1:400		abcam	A6721
